# Delayed Intracranial Hemorrhage in Patients with Head Trauma and Antithrombotic Therapy

**DOI:** 10.3390/jcm8111780

**Published:** 2019-10-25

**Authors:** Anna Antoni, Elisabeth Schwendenwein, Harald Binder, Martin Schauperl, Philip Datler, Stefan Hajdu

**Affiliations:** 1 Department of Orthopedics and Trauma-Surgery, Medical University of Vienna, 1090 Vienna, Austria; elisabeth.schwendenwein@muv.ac.at (E.S.); harald.binder@muv.ac.at (H.B.); martin.schauperl@muv.ac.at (M.S.); stefan.hajdu@muv.ac.at (S.H.); 2 Department of Anesthesia, Intensive Care Medicine and Pain Medicine, Medical University of Vienna, 1090 Vienna, Austria

**Keywords:** delayed intracranial hemorrhage, head trauma, traumatic brain injury, antithrombotic therapy

## Abstract

Background: Delayed intracranial hemorrhage can occur up to several weeks after head trauma and was reported more frequently in patients with antithrombotic therapy. Due to the risk of delayed intracranial hemorrhage, some hospitals follow extensive observation and cranial computed tomography (CT) protocols for patients with head trauma, while others discharge asymptomatic patients after negative CT. Methods: We retrospectively analyzed data on patients with head trauma and antithrombotic therapy without pathologies on their initial CT. During the observation period, we followed a protocol of routine repeat CT before discharge for patients using vitamin K antagonists, clopidogrel or direct oral anticoagulants. Results: 793 patients fulfilled the inclusion criteria. Acetylsalicylic acid (ASA) was the most common antithrombotic therapy (46.4%), followed by vitamin K antagonists (VKA) (32.2%) and Clopidogrel (10.8%). We observed 11 delayed hemorrhages (1.2%) in total. The group of 390 patients receiving routine repeat CT showed nine delayed hemorrhages (2.3%). VKA were used in 6 of these 11 patients. One patient needed an urgent decompressive craniectomy while the other patients were discharged after an extended observation period. The patient requiring surgical intervention due to delayed hemorrhage showed neurological deterioration during the observation period. Conclusions: Routine repeat CT scans without neurological deterioration are not necessary if patients are observed in a clinical setting. Patients using ASA as single antithrombotic therapy do not require in-hospital observation after a negative CT scan.

## 1. Introduction

While numerous guidelines for the management of mild traumatic brain injury (TBI) exist, there is still controversy regarding the treatment of head trauma patients with antithrombotic therapy (ATT). The management of mild TBI on ATT is complicated by the heterogeneity of patients with different medications and varying patient characteristics. Most authors define mild TBI based on a Glasgow Coma Scale (GCS) of 13 to 15, others include any impact to the brain, not necessarily causing symptoms [1]. For patients using ATT, several studies show an increased risk for abnormal computed tomography (CT) findings, even with a normal neurological exam and a history lacking neurological symptoms [2,3,4]. Therefore, in most centers patients on ATT receive a routine CT at presentation, even if common definitions of mild TBI are not fulfilled and head trauma is merely reported, or visible signs of head trauma are present.

Vitamin K antagonists (VKA) were shown to increase the risk for clinically significant TBI and mortality [5,6,7] and numerous studies indicate an increased risk and mortality for patients on all other kinds of ATT [8,9,10,11,12].

Delayed traumatic intracranial hemorrhage (DIH) can occur up to several weeks after trauma to the head [13] and was reported to occur more frequently in patients with ATT, ranging from 0.2% to 6% [14,15,16,17].

Due to studies showing a high number of DIH, international guidelines and recommendations in literature suggest admitting patients with ATT for observation after negative CT [18,19,20,21]. Many trauma centers adopted management protocols highly cautious of DIH and performed repeat CT after head trauma for asymptomatic patients. These extensive management protocols with in-hospital observation and repeat CT were evaluated in numerous studies, and most authors concluded that a routine repeat CT is not necessary [22,23]. Some authors even question the necessity for clinical observation after negative CT [18,24,25].

## 2. Materials and Methods

Our level I trauma center follows a high level of precaution for head injury patients. CT are performed based on the Canadian CT Head Rule, which, however, excludes patients with ATT [26]. At the time of data collection, all cases of head trauma with ongoing ATT regardless of clinical signs for TBI received a CT and were admitted for a minimum of 24 h of in-hospital observation. Patients receiving VKA, Clopidogrel or direct oral anticoagulants (DOAC) received a routine repeat CT before discharge to detect delayed hemorrhages. Patients using acetylsalicylic acid (ASA) or low molecular weight heparin (LMWH) underwent clinical observation but did not receive a routine repeat CT.

After two years of said management, we analyzed our clinical protocol to determine the frequency of delayed intracranial hemorrhage in patients with head trauma and antithrombotic therapy, adjusting our practice and thereby contributing to the ongoing international debate on the management of head trauma patients on ATT.

The study was performed in a level I trauma center with authorization by the local Institutional Review Board (1632/2014). Between January 2012 and April 2014 patients aged 18 years or older were retrospectively included if they were admitted for observation after blunt head trauma with ongoing ATT and no pathologies in their initial CT. Management of these patients followed the described standard clinical protocol. This included an initial CT, clinical and GCS assessment including history of unconsciousness and laboratory tests including S100 and coagulation studies at time of admission. We did not routinely perform laboratory tests for evaluation of therapeutic levels during the observation period (viscoelastic tests, platelet function or anti-Xa assays) for ATT other than VKA. In-hospital observation for a minimum of 24 h followed, and patients received their applicable protocols:

- Patients using ATT with an expected higher risk for DIH based on the literature, such as vitamin K antagonists (VKA), direct oral anticoagulants (DOAC) and Clopidogrel received a routine repeat CT prior to discharge from hospital.
- Patients using ATT with an expected low risk for DIH including acetylsalicylic acid (ASA) and prophylactic doses of low molecular weight heparin (LMWH) did not receive a routine repeat CT and were discharged after observation only. Due to the greater number of patients receiving ASA compared to other ATT we included only patients from January 2013 until December 2013 in this study.

The primary endpoint of this study was the occurrence of delayed intracranial hemorrhages. Data was collected and analyzed using SPSS version 24 and descriptive statistics were performed. Due to the low number of delayed intracranial hemorrhages the variables age, GCS and prothrombin time were tested using the Mann–Whitney–U test while the remaining nominal variables were tested using Fisher’s exact test. The significance level alpha for all implemented tests was set to α < 0.05.

## 3. Results

During the study period 793 patients fulfilled the inclusion criteria, with a majority of 453 (57.1%) women and 340 (42.9%) men. A routine repeat CT was performed in 395 cases and in-hospital observation without routine repeat CT in 398 patients. The average patient age at presentation was 81 years (range 32–102). The most prevalent ATT was acetylsalicylic acid in 368 patients (46.4%), followed by vitamin K antagonists in 255 (32.2%) and Clopidogrel in 86 patients (10.8%), see Table 1. Since patients using ASA were included from only one year whereas all other patients were collected from a two-year observation period, the distribution of different types of ATT is not representative of the total population at our institution.

Only blunt trauma was included in the study, with low energy trauma due to falls accounting for 95.2% of all cases. Most patients presented with a normal neurological status, only 16.5% of patients showed any neurological symptoms at presentation. The average GCS at presentation at the hospital was 15 and in 75.5% there was no history of unconsciousness or amnesia reported by either the patients or others. Lesions to the head such as abrasions and lacerations were present in 57.4%. The mean prothrombin time of the 255 patients using VKA was 32%, and 91.8% of the VKA patients were in their therapeutic range at time of admission.

The timing of the routine repeat CT as well as discharge from hospital followed the clinical protocol and occurred after a minimum observation time of 24 h, which resulted in an average of two nights of in-hospital observation, depending on the time of admission, with discharge after morning rounds.

In total, there were 11 cases (1.2%) of delayed intracranial hemorrhages. The routine repeat CT group showed nine DIH, resulting in 2.3% of cases detected with routine repeat CT. In the observation-only group, 16 patients showed a worsening of GCS or other symptoms indicating TBI. Two of these repeat CTs based on clinical judgement found minor DIH (0.5% of the observation group). One of the 11 patients with DIH needed an urgent decompressive craniectomy due to subdural hematoma with midline shift on the second day of observation. This was an 84-year-old female with vitamin K antagonist therapy, who showed no clinical signs of traumatic brain injury, no exterior injury to the head and an international normalized ratio of 2.9 at admission. The repeat CT was performed due to neurological deterioration with reduced Glasgow Coma Scale 27 h after admission. The patient underwent immediate decompressive craniectomy and was consecutively discharged to a neurological rehabilitation facility with a mild left-sided hemiparesis. The other patients with small delayed intracranial hemorrhage did not undergo surgical intervention and were discharged from hospital after an average observation period of 12 days (range 5–23). A review of the cases of delayed intracranial hemorrhages in the repeat CT group by a radiologist revealed, that small epi- and subdural hematomas, minimal intracerebral and subarachnoid hematomas were visible but not described in the initial CT report in four of the eleven cases. Excluding the four cases of initially undiagnosed pathologies in the CT report, an adjusted number of seven DIH (1.8%) were found in the repeat CT group, and in 0.9% overall. There were no significant differences between patients with or without delayed intracranial hemorrhage regarding age, sex, mechanism of injury, extent of external head injury, S100 level and coagulation studies or neurological status at admission. Characteristics of patients with DIH in our study population can be seen in Table 2. Four patients died during the in-hospital observation, all due to non-TBI-related causes such as pneumonia or heart failure.

## 4. Discussion

No feasible diagnostic or observation protocol will be able to exclude all cases of fatal delayed bleeding as DIH can occur up to several weeks after head trauma. Most clinically significant DIH will be detected within an observation period of 24 h, but there will always be cases that occur unusually late or in surprisingly neurologically intact patients [14,15,16,17,19,20,21].

The results of our study, with only one clinically significant case of delayed intracranial hemorrhage (0.3% in the repeat CT group, 0.1% in total), support other investigations which concluded that a routine repeat CT is not necessary for patients with antithrombotic therapy, due to the low clinical significance of most detected DIH [27,28,29,30,31,32]. The single case of a clinically significant DIH in our study showed an altered neurological status and would therefore have received an additional CT during the observation period regardless of clinical protocol for routine repeat CT. In our study population the routine repeat CT was effective in detecting DIH, but was superior to clinical observation only for the detection of cases of little clinical significance and devoid of therapeutic consequence.

Based on our results and on the growing international consensus eliminating repeat CT, we have changed our clinical protocol to a 24-h observation period after the initial negative CT, and discharge from hospital without repeat CT. Due to the low number of DIH in the observation group using ASA and/or LMWH with 0.5%, we no longer admit these patients for in-hospital observation after a negative CT. Since we only included patients using ASA from one year compared to a two-year observation period for all other ATT, the total impact of not routinely admitting these patients after a negative CT is even greater, uses less resources and can improve patient satisfaction.

Most studies addressing the management of head injury patients on ATT do not explicitly discuss the degree of head injury. Most patients in our study would have not received a CT based on the commonly applied Canadian CT Head Rule had they not been treated with ATT, as the majority of patients had a normal neurological exam at presentation and only 57.4% of patients in our study had visible signs of a head injury. Because of the potential higher risk for intracranial pathologies in patients with all types of ATT we support the recommendation of performing an initial CT as suggested by the Scandinavian Guidelines [18] and the recent Austrian consensus statement [33]. However, the increasing number of patients with ATT in a time of limited resources raises questions about a clear definition of head trauma to determine risk factors for DIH. Whether a reported minor impact to the head without visible injuries justifies the cost and patient inconvenience of enduring a CT and in-hospital observation cannot be answered by our study and remains a decision based on clinical judgement.

Despite all guidelines, it is still necessary to make decisions based on risk stratification while considering the consequences for the individual patient. Chenoweth et al. [28] found a 0.3% rate of DIH in their prospective study including patients with and without ATT, and concluded that “this (the low risk of DIH and fact that they can occur later than 24 h) highlights the importance of clinical judgment regarding the severity of trauma, additional injuries, and ability to monitor the patient for deterioration when making decisions about admission for older patients after blunt head trauma.”

However, our patient requiring surgical intervention, while only representing 0.1% of our study population, initially showed no clinical signs of a massive trauma to the head and her only risk factors for significant TBI were her age and her vitamin K therapy. This patient could have died at home if she were discharged without observation by relatives or nurses after the initial CT. 

Clearly, the admission of patients must be indicated based on economic factors as well as on aspects of the patient’s will and ethical considerations for elderly patients, who might experience massive stress due to the changed environment in the hospital [34]. A relevant but unanswered question underlying this study is, how does the fear of legal consequences affect clinical management, although a potentially fatal DIH may not lead to an escalation of care due to advanced patient age? If a patient admitted for observation were to incur a DIH, would the same patient be operated on if he or she was 95 years old, not living independently and suffering from dementia?

The average age of patients in our study was 81 years. Unfortunately, data on dementia, degree of dependency or existence of a living will was not available for our retrospective study design. 

In our study some 91.8% of patients using VKA were in a therapeutic range and we did not perform further coagulation assays specific to other ATT during the observation period. Assessing additional therapeutic effects of ATT at time of presentation could possibly reduce admissions and save resources, while informing patients of their insufficient hypocoagulative protection.

Limitations of the study are explained by the retrospective study design and lack of data about DIH after discharge. Furthermore, the number of patients using DOAC was relatively low at the time of data collection but is now a controversial topic in literature. Since there may have been cases of undetected DIH in the group without routine repeat CT and the low number of patients using DOAC this study cannot address the relative risk of various ATT for DIH. The large number of patients and relative homogenous population constitute a significant strength of our study, but multicenter prospective trials are needed to further investigate this matter. 

Consistent with current studies, our results indicate that routine repeat CT seem to be no more effective than in-hospital observation, due to the fact that clinically significant DIH reveal themselves by neurological deterioration. Furthermore, we conclude that in-hospital observation for head trauma patients using acetylsalicylic acid is not necessary due to the rarity of clinically relevant DIH. But neither our study nor current literature can answer the ethical questions behind the data. They must be answered by individual centers and countries based on existing resources and their cultural environment.

## Figures and Tables

**Table 1:** Type and frequency of antithrombotic therapy in total and in the subgroups of the study.

Antithrombotic Therapy	Total n (%)	Repeat CT Group n (% of Group)	Observation Group Only n (% of Group)
Acetylsalicylic acid	368 (46.4%)	0	368 (92.5%)
Vitamin K antagonists	255 (32.2%)	255 (64.6%)	0
Clopidogrel	86 (10.8%)	86 (21.8%)	0
Clopidogrel and acetylsalicylic acid	22 (2.8%)	22 (5.6%)	0
DOAC (dabigatran and rivaroxaban)	32 (4.0%)	32 (8.1%)	0
Low molecular weight heparin	26 (3.3%)	0	26 (6.5%)
Low molecular weight heparin and acetylsalicylic acid	4 (0.5%)	0	4 (1.0%)

**Table 2:** Characteristics of patients with delayed intracranial hemorrhage in our study population including sex and age, antithrombotic therapy, reported unconsciousness and amnesia, prothrombin time at admission, neurological symptoms during in-hospital observation, type of delayed intracranial hemorrhage (EDH = epidural hematoma, SDH = subdural hematoma, SAH = subarachnoid hematoma, ICH = intracerebral hematoma) and necessity of surgery. ASA = acetylsalicylic acid.

Patient	Antithrombotic Therapy	Unconsciousness	Amnesia	Glasgow Coma Scale	Head Wound	Prothrombin Time	Neurological Symptoms	Delayed Intracranial Hemorrhage	Surgery
m, 72y	Vitamin K antagonist	yes	yes	15	yes	56	no	EDH, SDH	no
f, 93y	Vitamin K antagonist	no	no	15	yes	47	no	SAH	no
f, 83y	Vitamin K antagonist	no	no	15	yes	20	no	SAH	no
f, 82y	Vitamin K antagonist	no	no	15	yes	48	no	SAH	no
m, 92y	Vitamin K antagonist	no	no	15	yes	33	no	SAH	no
f, 84y	Vitamin K antagonist	no	no	15	no	27	yes	SDH	yes
m, 90y	Clopidogrel + ASA	no	no	15	yes	89	no	Hygroma	no
m, 54y	Clopidogrel + ASA	no	yes	15	yes	105	no	ICH	no
m, 89y	Dabigatran	no	no	15	yes	89	no	SDH	no
f, 82y	ASA	no	no	15	yes	104	no (repeat CT was recommended by radiologist after review of initial CT)	EDH	no
f, 79	ASA	yes	yes	14	yes	88	yes	EDH	no
